# COVID-19 and Black, Asian, and Minority Ethnic Communities: A Complex Relationship Without Just Cause

**DOI:** 10.2196/22581

**Published:** 2021-02-01

**Authors:** Peter Phiri, Gayathri Delanerolle, Ayaat Al-Sudani, Shanaya Rathod

**Affiliations:** 1 Research & Development Department Tom Rudd Unit, Moorgreen Hospital Southern Health NHS Foundation Trust Southampton United Kingdom; 2 University of Southampton Primary Care, Population Sciences and Medical Education, Faculty of Medicine Aldermoor Health Centre Southampton United Kingdom; 3 Oxford Brain Health Clinical Trials Unit, Department of Psychiatry Warnford Hospital University of Oxford Oxford United Kingdom

**Keywords:** BAME, COVID-19, ethnicity, health care professionals, health care worker, impact, inequalities, minority, risk

## Abstract

Emerging evidence has indicated a negative and disproportionate impact of COVID-19 on Black, Asian, and minority ethnic (BAME) communities. Previous studies have already reported that biological and social risk factors increase disease susceptibility, particularly in BAME communities. Despite frontline workers in ethnic minority communities in the United Kingdom’s National Health Service attempting to quell the pandemic, disproportionate numbers of BAME physicians and other health care workers have died of COVID-19. This unprecedented situation highlights ethical and moral implications, which could further augment the impact of the pandemic on their mental health. While the government attempts to mitigate the rate of virus transmission, certain key factors inadvertently augment the negative impact of the pandemic on the mental health and general well-being of BAME communities. This study examined the available literature to explore the association between, and the wider impact of, COVID-19 on BAME communities. Furthermore, this study aims to raise awareness and provide a deeper insight into current scientific discussions.

## The Disproportionate Impact of COVID-19 on Black, Asian, and Minority Ethnic Communities

The first 10 health care professionals in the United Kingdom to die of COVID-19 were from the Black, Asian, and minority ethnic (BAME) communities. This raised concerns of a potential association between ethnicity and a disproportionate impact of COVID-19 on these communities [1-3]. The Intensive Care National Audit and Research Centre published UK data sets from April 10, 2020, showing that one-third of COVID-19 patients admitted to critical care units belonged to an ethnic minority group. Among 3883 COVID-19 patients, 14% (n=486) were Asian and 12% (n=402) were Black [4]. Similarly, *The Guardian* [5] reported that among 12,593 patients, 19% (n=2393) of those who died of COVID-19 in hospital up till April 10, 2020, were from BAME communities. These data are discordant to those of BAME individuals in the general population in the United Kingdom (14%) [1,6]. In addition, *The Washington Post* [1] reported that in the United States, Black-majority counties have 3-fold the number COVID-19 cases and almost 6-fold the number of COVID-19–related deaths compared to White-majority counties. However, caution should be exercised when generalizing across different health care systems [1]. A Public Health England (PHE) report [7] acknowledges the disproportionate impact of COVID-19 on BAME communities, reporting increased mortality, despite the absence of measures to potentially address the concerns identified therein.

## Deaths Among National Health Service Health Care Professionals in BAME Communities

On March 12, 2020, chief medical officers in the United Kingdom elevated the country’s risk status from moderate to high, and on March 23, 2020, the prime minister imposed a nationwide lockdown [8]. Since then, 181 National Health Service (NHS) workers have died of COVID-19 [9]. This figure, however, mainly relies on reports from hospitals within England only. Numerous reports have claimed that approximately 62%-75% of COVID-19–related deaths among health care workers occurred in BAME workers [1,2,4]. This is alarming because only one-third of NHS physicians in hospitals or community services in England belong to Asian (27.2%) or Black (6.95%) communities [10].

## Disease Susceptibility and Predictability in BAME Communities

Many experts, including Duncan Young (professor, Intensive Care Medicine, University of Oxford), Dr Riyaz Patel (associate professor, Cardiology, University College London), and Naveed Sattar (professor, Metabolic Medicine, University of Glasgow), have suggested that ethnic minorities are at an increased risk of SARS-CoV-2 infection, severe disease, and poor outcomes owing to socially and biologically relevant reasons [11]. First, ethnicity could play a major role in disease transmission owing to cultural, behavioral, and societal differences including those in health-seeking behaviors [12], cohabiting lifestyle [13], and lower socioeconomic status. The disease transmission risk is further increased among NHS workers. Furthermore, individuals in ethnic minority communities are disproportionally employed in fields including those associated with public transport or delivery services, where there is a known, markedly higher risk of virus transmission. It is also common for BAME households to have several generations cohabiting within close confinement as culture and family are potentially important aspects of identity in these communities. Thus, it could be challenging for BAME communities to follow social distancing protocols [14,15]. Furthermore, complexities in other comorbidities such as diabetes, hypertension, and cardiovascular diseases are commonly associated with South Asians [11]. Another theory is based on the similarities observed between the risk of morality during the last major influenza crisis—the H1N1 epidemic—and ethnic minority communities in 2009-2010 and during the first postpandemic season of 2010-2011 in England. From this data set, 67 of 337 (19.9%) individuals were from BAME communities. Furthermore, ethnic minorities have been at a higher mortality risk than the Caucasian population during the 2009-2010 pandemic, with individuals of Pakistani descent being at the highest risk [16].

In addition, early studies on the disproportionate prevalence and severity of respiratory diseases among BAME communities suggest predictable health outcomes based on socioeconomic status [17]. Social stressors and environmental adversity appear to be linked to an elevated risk of cardiovascular disease and other comorbidities [16]. According to Carol Cooper, the head of equality, diversity and human rights at Birmingham Community Healthcare NHS Trust [18]:

Many of us knew that BAME people would be overrepresented - given their proportion of the population - in the mortality and morbidity figures because of the comorbidities that exist in our communities, because of the location of our communities in terms of the workforce being on the frontline [and] because of the amount of people that are caught in the poverty trap and live in households that have higher occupancy.

Despite previous warnings and the need for public health authorities to identify at-risk populations, a literature review indicated that only 2 of 29 (7%) publications reported disaggregated data on ethnicity (case series without ethnicity-specific outcomes) [13]. The countries that initially reported the highest number of COVID-19 cases did not report data on ethnicity [13]. Researchers in the United Kingdom did not acquire or publish information on ethnicity until concerns of an association between COVID-19 and ethnicity began to emerge [3,13,19]. As is the case in many diseases, researchers and policy makers do not often consider ethnicity as a core factor until deaths among BAME communities become prominent in mortality data or media reports.

In addition to reports from the United Kingdom [11], those from the United States have indicated that chronic conditions such as diabetes, asthma, hypertension, kidney disease, and obesity are more common in Black American than in White American populations [20]. These conditions are associated with poor outcomes in COVID-19 cases. Moreover, Kirby [20] reported:

The risks of COVID-19 to Indigenous communities could not be clearer. More than 1 in 3 Indigenous Australian adults report having either cardiovascular disease, diabetes, or renal disease, and onset of these diseases often occurs 20 years earlier than the non-Indigenous population.

The NHS Long Term Plan has identified and prioritized more common conditions including diabetes, hypertension, and obesity, but has overlooked other, more specific health conditions that increase disease severity in BAME communities, such as asthma and kidney and cardiovascular diseases [21,22]. Similarly, these conditions do not seem to be prioritized by US health care authorities [23].

## Inequalities and Their Psychological Impact

Stress-related physiological and general psychological responses, such as recurrent experiences of discrimination, can significantly impact health by increasing the risk of heart disease, diabetes, and infections [24]. The PHE report of 2020 [22] asserts that this pandemic did not generate health inequalities but merely exposed and exacerbated the longstanding health and socioeconomic inequalities affecting BAME communities in the United Kingdom. Although this statement might hold true, perceptions of the underlying causal relationships vary greatly and are difficult to unravel [7]. Compared to the United Kingdom, hate crimes against Asian Americans have increased in the United States [25]. A study from the Healthforce Center at the University of California San Francisco [25] reported that 25% of working nurses are either Asian-born or Asian American in California alone. In the United Kingdom, over 1 in 5 allied health professionals, such as nurses, health visitors, and midwives, belong to a BAME community [26]. Similarly, Cook et al [27] reported that among 119 NHS deaths recorded in the NHS staff, 35 occurred among nurses, 27 among health care workers, and 18 among physicians. Furthermore, nurses belonging to BAME communities are more likely to report higher levels of stress and show signs of posttraumatic stress or other common mental health disorders than their non-BAME counterparts [28]. Furthermore, social media platforms reporting these stories can potentially influence global communities [29].

In an interview with *Nursing Times*, Carol Cooper added, “BME staff feel that they are being put on Covid wards and exposed to patients with Covid over and above their colleagues” [18]. The NHS Staff Survey of 2020 and data from the NHS Workforce Race Equality Standard (WRES) consistently provide evidence on staff in BAME communities, reporting instances of discrimination, harassment, and victimization from other staff members and the general public [26]. Consequently, some NHS staff from BAME communities may not feel confident in requesting necessary items such as personal protective equipment (PPE) and COVID-19 tests to ensure their safety. Feedback from BAME staff [14] also suggested that some forms of PPE may not be suitable. For example, some Muslim health care professionals wear a head covering (referred to as a “hijab”); hence, wearing face masks and visors may be difficult. In addition, some Muslim and Sikh men may have a beard, which could also affect the fit of face masks or PPE. Furthermore, key measures or restrictions to prevent the spread of infection could potentially be more hazardous to ethnic minorities than to others, such as withdrawing key services, implementing no-visitor policies, and social isolation or quarantine [14]. Without translation or language support, some patients are unable to articulate their health needs. Isolation is difficult owing to the multigenerational households in these communities. Furthermore, the closure of religious or community centers can impact psychological well-being. Compared to nonattenders, individuals from BAME communities who visit religious places of worship or community centers presented reduced suicide rates [30]. Closure of churches, community centers, and mosques can therefore result in poor mental health; this can, in turn, affect physical health, thus reducing the chances of survival among individuals of BAME communities [24,31].

In addition, Greenberg et al [32] reported that health care professionals are at an increased risk of “moral injury” and mental health difficulties. Health care workers are faced with detrimental decision making and extreme pressures both before and during the pandemic. Health care workers must be able to ensure the welfare of both themselves and others. They must maintain a balance between “desire” and “duty,” while working with insufficient resources, particularly during COVID-19 [32]. Furthermore, Greenberg et al [32] reported that individuals developing moral injuries were likely to have negative thoughts about themselves or others as well as feelings of shame, guilt, or disgust. Negative thoughts can often lead to the development of common mental health problems, including depression and posttraumatic stress disorder, in turn affecting physiological health and even leading to suicidal ideation.

Health workers in Pakistan who have been under physical and psychological pressure have seen an increased incidence of mental health symptoms, such as heightened fears and anxiety, which could have long-term and detrimental effects on overall well-being [28]. Rana et al [28] suggested that intervention might involve the development and delivery of online content by mental health professionals to spread awareness of the psychological impact of pandemics. Psychological factors such as fear and prejudices associated with COVID-19 have led to notable levels of xenophobia, and this might have led to Zahidul Islam—a 36-year-old Bangladeshi man—committing suicide on March 25, 2020 [33,34]. This again may support the negative effects of closing religious and community centers or other places of importance, and social isolation. Zahidul may have also believed it was his “moral duty” not to transmit the virus, although his tests revealed that he did not harbor the infection [34].

Accordingly, researchers from National University Health System and Yong Loo Lin School of Medicine used a self-administered questionnaire to examine psychological distress, depression, and anxiety among health care workers in Singapore [35]. Tan et al [35] reported that during the peak of the pandemic, the incidence of anxiety was increased among nonmedical health care workers, probably owing to “less first-hand medical information on the outbreak and less intensive training on PPE and infection control measures.” These suggestions are consistent with those of a systematic literature review [36] wherein numerous studies highlighted the importance of preparation, including training and work experience, during a crisis. Brooks et al [37] aimed to identify the social and occupational factors affecting the psychological well-being of health care workers involved in the severe acute respiratory syndrome crisis. They reported that “those who perceived their training as inadequate were more likely to experience burnout, posttraumatic stress and longer continuing perceived risk” [37]. This may be why death rates among NHS workers in BAME communities have been so high, and the WRES [26] similarly indicated that White NHS workers are more likely to have greater access to nonmandatory training and continuous professional development than their BAME counterparts. Thus, White people may be better informed and therefore better able to cope with pandemics than their BAME counterparts.

**Figure 1:**
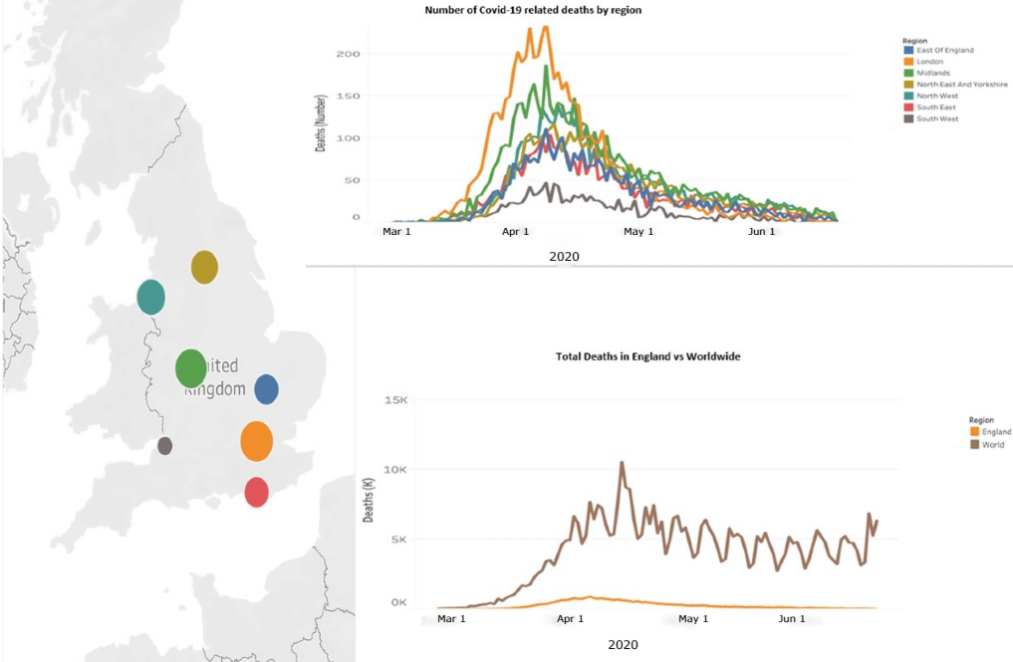
Total COVID-19-related deaths at National Health Service Hospitals in England (data up to June 19, 2020).

**Figure 2:**
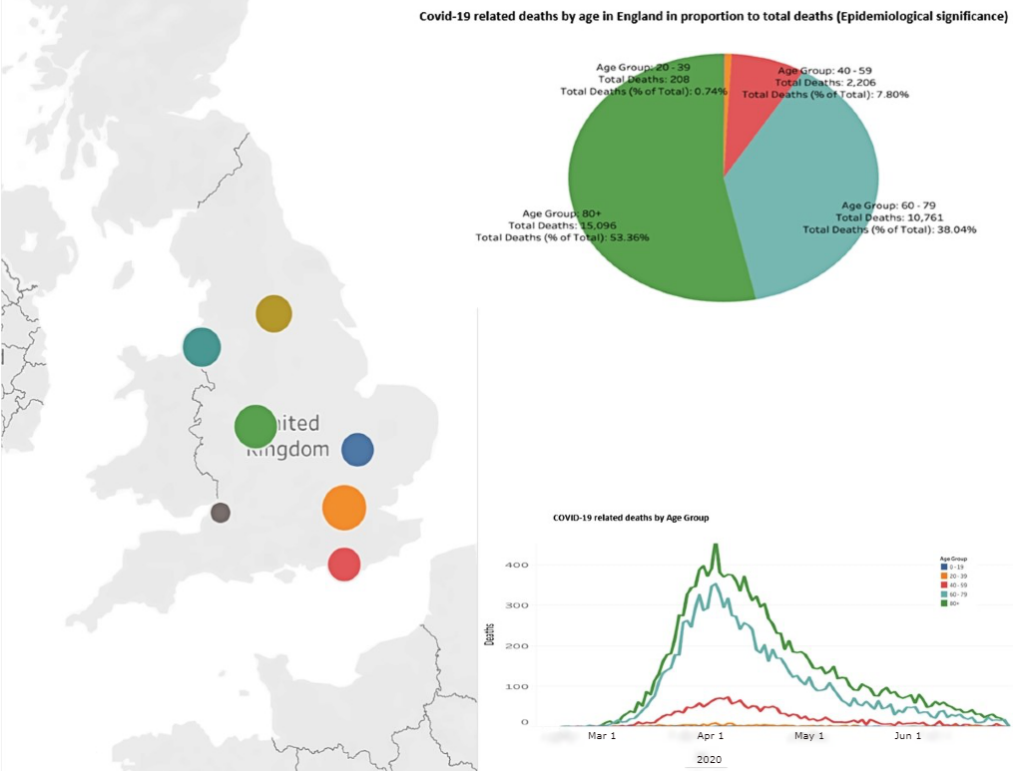
COVID-19-related deaths by age in England (data up to June 19, 2020).

The COVID-19 pandemic has threatened the health and lives of millions of individuals worldwide, and data from the Johns Hopkins University have reported 38,272,349 confirmed cases and 1,088,051 global deaths as of June 30, 2020 [38]. As of October 2, 2020, the United Kingdom has reported 42,369 deaths [39]. Figures 1 and 2 show the total deaths at NHS hospitals in England. As such, a virus may not discriminate among individuals; however, society apparently does, as emphasized in a recent editorial [40].

Negative effects of government measures to mitigate virus transmission albeit unintentionally increase health inequalities, including mental health inequalities [14,24,30,31]. Inequalities in wages and career development in the NHS, combined with discrimination at work and in society, restrict BAME workers to certain roles potentially rendering their specialties and services critical for combatting COVID-19 and other pandemics [40]. As previously indicated, health care workers in BAME communities may not have the confidence to voice their concerns regarding inadequate PPE, long hours of work, and low wages, which unnecessarily places them at a higher risk of succumbing to the infection [40]. Even at higher-level positions, BAME physicians and health care workers are perhaps as vulnerable as those at lower socioeconomic positions [40]. Stress, inequality, trauma, discrimination, and marginalization can negatively impact the working environment of individuals belonging to BAME communities. Furthermore, inadequacies apparent within the research sphere fail to address the inequalities among BAME groups. This was observed even during past epidemics such as the H1N1 influenza (2009), polio (2014), Ebola in West Africa (2014), Zika (2016), and Ebola in the Democratic Republic of the Congo (2019) [27], and notable effects were also observed among individuals in BAME communities in the United Kingdom [16]. Inequalities in addressing biological or physiological risk factors will inevitably lead to poor outcomes among ethnic minorities than among other populations [11,40].

## Conclusion

Emerging data strongly suggest a disproportionate impact of COVID-19 on BAME communities in the United Kingdom [1-11]. A disproportionate number of BAME physicians and health care workers in the NHS have died during the pandemic [9,10]. Nonmedical staff are at the highest risk of psychological distress, while health care professionals from BAME communities are more likely to succumb to the pandemic owing to discrimination and inequalities both at the workplace and in society [35]. These effects are further bolstered by recent government measures such as social isolation, social distancing, and lockdowns to prevent virus transmission. Stress-related physiological and psychological responses can significantly affect health by increasing the risk for heart disease, diabetes, and infection; this is a primary biological explanation for the aforementioned disproportionate impact [11]. Although mental health problems may be a secondary outcome of the pandemic, they need to be a primary focus area owing to their longer-lasting effects than those of COVID-19. The crisis thus presents an opportunity to improve mental health and bridge the aforementioned inequality gap [40]. More evidence, research, and global data on ethnicity are needed to confirm speculations of conjectured associations between COVID-19 and BAME communities, and to inform these communities about the current policies and practices. This is particularly important for regions where the pandemic is at an early stage or in those preparing for a second wave of infection. While the United Kingdom may have been late in preventing virus transmission compared to other countries, psychological intervention need not be delayed.

## Abbreviations

BAME: Black, Asian, and minority ethnic
NHS: National Health Service
PHE: Public Health England
PPE: personal protective equipment
WRES: NHS Workforce Race Equality Standard
